# Supplementary material for: Piezo robotic hand for motion manipulation from micro to macro
Source: Nat Commun. 2023 Jan 30;14:500. doi: 10.1038/s41467-023-36243-3 (PMC9887007; doi:10.1038/s41467-023-36243-3)
Supplement: Supplementary file 1 — Supplementary Information [file 41467_2023_36243_MOESM1_ESM.pdf]

Supplementary information for

Piezo robotic hand for motion manipulation from micro to macro

Shijing Zhang†[1], Yingxiang Liu†*[1], Jie Deng†[1], Xiang Gao[1], Jing Li[1], Weiyi Wang[1], Mingxin Xun[1], Xuefeng Ma[1], Qingbing Chang[1], Junkao Liu[1], Weishan Chen[1], Jie Zhao[1]

Affiliations:

[1]State Key Laboratory of Robotics and System, Harbin Institute of Technology; Harbin 150001, China.

†These authors contributed equally to this work.

*Corresponding author. Email: liuyingxiang868@hit.edu.cn

This PDF file includes:

Supplementary Note 1 to Note 10.

Supplementary Table 1 to Table 3.

Supplementary Fig. 1 to Fig. 8.

Supplementary references.

Other supplementary materials for this manuscript include the following:

Supplementary Movies 1 to 10

Supplementary Note 1

Motion forms, DOFs and supporting strategy of the manipulated objects

In this work, we expect to manipulate various objects including the flatbed, cylindrical and spherical ones to achieve multi-DOF motions. As for different objects, they hold diverse motion forms and DOFs (Supplementary Fig. 1a to Fig. 1c). For the convenience of description, the coordinate system XYZ is defined on the geometric center of the three kinds of objects, as well as we utilize the labels LX, LY, and LZ to indicate linear motions along X, Y, and Z axes, respectively; similarly, the labels RX, RY, and RZ are used to indicate rotary motions around X, Y, and Z axes, respectively. Accordingly, the typical motion DOFs of the flatbed, cylindrical and spherical objects can be represented by 3L+3R DOFs (LX, LY, LZ, RX, RY, and RZ), 1L+1R DOFs (LX and RX) and 3R DOFs (RX, RY and RZ), respectively.

One of our design ideas is to integrate the manipulating ends and supporting ends: the fingertips of the PRH are not only used to support the manipulated objects, but also to be the manipulating ends. We use four supports to support the three kinds of objects, in which the four supports are uniformly distributed at the four corners of a square (Supplementary Fig. 1d to Fig. 1f). The advantage of this supporting strategy is to ensure the symmetry of the supporting relationships of the three kinds of objects in XOY plane. The expected motions of the three kinds of manipulated objects can be achieved by the collaborative manipulation of the four piezo fingers through frictional actions.

Supplementary Note 2

Configurations and deformation principles of the actuation part

The actuation part can be utilized to generate three dimensional deformations for further inducing motions of the whole piezo finger. The detailed structure, electrical configuration and

fundamental principles of the actuation part are as follows. (i) The actuation part is composed of 10 groups of basic actuation units, and each actuation unit includes two piezoelectric ceramic rings, a common electrode and a group of fan-shaped electrodes (Supplementary Fig. 2a). These electrodes are designed to apply electric field between the two surfaces of the piezoelectric ceramic. (ii) The whole motions of the piezo fingers are induced by accumulating micro deformations of the actuation units, depending on specific polarization and placement of the piezoelectric ceramic. Therefore, we present two adjacent piezoelectric ceramics in one actuation unit to illustrate their polarization and placement relationships (Supplementary Fig. 2b). The piezoelectric ceramic rings are designed as four fan-shaped areas for polarizing respectively. In detail, the four areas are labeled as U_1, U_2, U_3 and U_4. A narrow gap is remained between the two adjacent fan-shaped areas to apply different electric fields on them. As indicated by the red arrows, each fan-shaped area is polarized along the thickness direction, and the polarization directions of the two nonadjacent fan-shaped areas are opposite. Besides, the two piezoelectric ceramic rings are arranged in spatial orthogonal. (iii) As for the electrical connections (Supplementary Fig. 2c), the common electrode is connected to the ground, which is defined as EG; the four fan-shaped electrodes are connected to EX+, EX-, EY+, and EY-, respectively. According to the special configurations of the two adjacent piezoelectric ceramics, the actuation unit can be excited to produce bending or extending deformations by applying electrical fields on the defined electrodes.

The deformations of the actuation part can be excited by the inverse piezoelectric effect of the polarized piezoelectric material. The detailed principles can be illustrated as follows: Considering a micro element of piezoelectric ceramic (Supplementary Fig. 2d), which is polarized along its thickness direction (see red arrow), if an electric field is applied between the

upper and lower surfaces (see blue arrow), it will produce extensional or contractional mechanical deformations (see magenta arrow) along the thickness direction, and the deformation direction depends on the electric field. This is also known as d_{33} working mode of the piezoelectric ceramic. As for the actuation part of the piezo finger, it can be regarded as a cylindrical body with piezoelectric characteristics and partitioned cross section (Supplementary Fig. 2e); according to the above structural and electrical configurations, the actuation part can adopt zonal polarization and excitation strategies to generate bending and extending motions as follows: (i) when the same exciting electrical field is applied to the partitions of 1 and 3 or the partitions of 2 and 4, the actuation part produces bending motion because that one side is compressed and the other side is extended; (ii) when one exciting electrical field is applied to the partitions of 1 and 2, and its reverting electrical field is applied to the partitions of 3 and 4 simultaneously, the actuation part can generate extending or contracting motion along the vertical direction (Supplementary Fig. 2f). To sum up, the actuation part can produce multi-dimensional bending and extending motions by accumulating basic deformations of ten groups of actuation units, this can be further used to induce the multi-dimensional motions of the whole piezo finger.

In fact, we can also use other polarization configurations and schemes to realize similar motions. A representative configuration is shown in Supplementary Fig. 2h to Fig. 2j. In detail, as shown in Supplementary Fig. 2h, two groups of piezo rings can be configurated to form an actuation part (divided as top half and bottom half), in which the top half and the bottom half contain several actuation unit-I and several actuation unit-II, respectively. The copper electrode slices are set between the adjacent two piezo rings to apply electric fields on them. It should be noted that every piezo ring in unit-I and unit-II holds two polarized regions, and the piezo rings

in unit-I and that in unit-II are configurated to mutually orthogonal (Supplementary Fig. 2i). With these configurations, the top half and the bottom half can separately produce 1-dimensional bending deformation when the same electric fields are applied on them simultaneously, while they can also produce 1-dimentisonal extending deformation when the opposite electric fields are applied on them simultaneously. Therefore, the whole actuation part integrating piezo rings with two polarized regions can produce bending deformations along x and y axes, and extending deformation along z axis (Supplementary Fig. 2j). It should be noted that this configuration will lead to response difference in the two bending deformations. This is because that there is an amplification effect (by the height of the top half of the actuation part) on the response when the bottom half of the actuation part is stimulated (assuming to excite bending deformation along x axis), whereas there is no amplification effect when the top half is stimulated (assuming to excite bending deformation along y axis). Therefore, in order to avoid the response difference in the two lateral bending motions of a piezo finger when using the same number of piezo rings to stimulate motions, the configurations of using piezo rings with four polarized regions are utilized in this work.

Supplementary Note 3

Material parameters of the PRH components

In terms of material configurations of the PRH components, the palm and the finger bases are stainless steel with good rust resistance (Density of 7.75×10^{3} kg/m^{3}, Young's modulus of 1.93×10^{11} N/m^{2}, and Poisson's ratio of 0.31); the common electrodes and the fan-shaped electrodes are set as beryllium bronze with good electrical conductivity (Density of 8.83×10^{3} kg/m^{3}, Young's modulus of 1.1×10^{11} N/m^{2}, and Poisson's ratio of 0.34); all of the used screws are standardized with stainless steel material; the fingertips are set as aluminum alloy (Density of

$2.77{\times}10^3$ kg/m^3, Young's modulus of $7.1{\times}10^{10}$ N/m^2, and Poisson's ratio of 0.33); the piezoelectric ceramics are made of PZT-4 (Lead Zirconate Titanate, Density of $7.6{\times}10^3$ kg/m^3). The more physical parameters of the PZT-4 are obtained from the supplier as follows:

$$[d] = \begin{bmatrix} 0 & 0 & 0 & 0 & 5 & 0 \\ 0 & 0 & 0 & 5 & 0 & 0 \\ -1.6 & -1.6 & 3.3 & 0 & 0 & 0 \end{bmatrix} \times 10^{-10}\,\mathrm{C/N} \tag{1}$$

$$[\varepsilon^T] = \begin{bmatrix} 8.1 & 0 & 0 \\ 0 & 8.1 & 0 \\ 0 & 0 & 6.7 \end{bmatrix} \times 10^{-9}\,\mathrm{F/m} \tag{2}$$

$$[c^E] = \begin{bmatrix} 14.3 & 7.85 & 7.85 & 0 & 0 & 0 \\ 7.85 & 14.3 & 7.85 & 0 & 0 & 0 \\ 7.85 & 7.85 & 11.5 & 0 & 0 & 0 \\ 0 & 0 & 0 & 2.6 & 0 & 0 \\ 0 & 0 & 0 & 0 & 2.45 & 0 \\ 0 & 0 & 0 & 0 & 0 & 2.45 \end{bmatrix} \times 10^{10}\,\mathrm{N/m^2} \tag{3}$$

where d, ε^T and c^E are the piezoelectric constant matrix, the dielectric matrix at constant stress and the stiffness matrix at constant electric field, respectively.

Supplementary Note 4

The influence of changing the number of the piezoelectric ceramic rings on the response displacement and response speed of the piezo finger.

We also analyzed the influence of the number of the piezo rings on the output displacement of the piezo finger by finite-element-method simulation with ANSYS. Meanwhile, the influence of the exciting voltage on the output displacement with different number of piezo rings is also simulated. Take the lateral bending motion as an example, the output displacement of the piezo finger is approximately linear with the number of the piezo rings (Fig. 2g), which means that the output displacement can be adjusted to meet some potential demands by changing the number of piezo rings. It should be noted that the simulation results in two bending motion directions are

same due to structural symmetry. Quantitatively, the simulated lateral output displacement of the piezo finger is about 24 μm under the maximum exciting voltage of 600 V_{p-p} when the number of the piezo rings are set as 20 PCS. When the number of the piezo rings increases to 2 times the situation used in this work (i.e., 40 PCS), the output displacement ascends to about 58.6 μm (relative to 2.44 times that under piezo rings of 20 PCS).

The influence of the number of piezo rings on the response speed can be analyzed qualitatively from the electrical-mechanical conversion processes. Theoretically, piezo rings are typical capacitance components, and they can produce response by two processes: (i) the exciting voltage from the external excitation devices is applied to the piezo rings to charge them; (ii) the piezo rings produce conversion of electrical energy to mechanical energy for producing deformations. The piezo rings theoretically need charging time t_1 and conversion time t_2 to complete these two processes, respectively. It should be noted that the charging time t_1 is more dominant than time t_2 due to the fast conversion characteristic of the piezoelectric ceramic, which means that the response time of the piezo rings mainly depends on the charge time t_1. As for the charge time t_1 of piezo rings, it mainly depends on the capacitance level of the piezo rings (determining how much energy needs to be charged) and the output current of the external excitation device (determining how fast to charge the energy). Single piezo ring is actually a parallel plate capacitance structure, and there is an electrical parallel relationship in multiple piezo rings. Therefore, the total capacitance will change when the number of the piezo ring changes, which affects the charge time t_1 and further affects the response speed of the piezo finger.

The capacitance level of the piezo rings can be described with mathematical expression as follows:

$$C_p = \sum_{i=1}^{n} \varepsilon \frac{S_i}{d_i} = n\varepsilon \frac{S}{d} \quad (4)$$

where, C_p is the total capacitance of one group of piezo rings used to generate 1-dimensioanl motion; n is the number of the piezo rings; S shows the relative electrode area of each piezo ring; d is the thickness of each piezo ring; ε is permittivity of piezo ceramic; it should be noted that the piezo rings are assumed to hold the same size (area S_i and thickness d_i) in the above equation.

The charging time of the piezo rings can be approximately expressed as follows:

$$t_1 \approx \frac{C_p U}{I} = n\varepsilon \frac{SU}{dI} \quad (5)$$

where, U and I are the exciting voltage applied on the piezo rings and output current of the external excitation device. The above equations indicate that the charging time t_1 is proportion to the number of piezo rings in theory. The total response time t can be represented as:

$$t = t_1 + t_2 \approx t_1 = n\varepsilon \frac{SU}{dI} \quad (6)$$

The conversion time t_2 can be ignored as it is short enough for piezoelectric ceramic, then the response speed can be regarded as only relation to charging time t_1. In another word, under the same external excitation conditions, changing the number of piezo rings with n times will cause an approximately n times change in response time.

In general, the average response speed can be regarded as the ratio between the output displacement and the response time. It should be noted that the increase of the number of the piezo rings n not only leads to the increase of the output displacement, but also causes the increase of the response time. Therefore, the influence of increasing the number of the piezo rings n on the response speed can be analyzed by estimating the increase level of the output displacement and the response time. (i) According to the above equation about response time t, if

the number of the piezo rings n increase to $2n$, the response time will increase to 2 times that corresponding to the number of n. (ii) According to the simulation results shown in Fig. 2g, when the number of the piezo rings n=20 increases to $2n$=40, the output displacement will increase to about 2.44 times of that corresponding to n=20. These results show that the change level of the output displacement is more than that of the response time when changing the number of piezo rings, which means that the increase of the number of piezo rings leads to the increase of the response speed in theory.

Supplementary Note 5

Characteristics of the piezo fingers

The fundamental characteristics of the piezo fingers just reflect the characteristics of the whole PRH, which determine the final manipulation performance. Therefore, we implemented several experiments to investigate the fundamental characteristics of the piezo fingers.

We investigated the input-output linearity of the four piezo fingers. The lateral bending motions of each piezo finger were excited by applying a sinusoidal signal with voltage of 600 V_{p-p} and frequency of 1 Hz, and the responding lateral displacements on the top of the fingertips were measured. Then the input-output characteristics, namely the relationships between the exciting voltages and the output displacements, were obtained (Supplementary Fig. 4a). The results show that the output displacements are linear with the exciting voltages, and there are slight nonlinear hysteresis effects caused by the inherent nonlinear characteristics of the piezoelectric materials (Supplementary Fig. 4b). Quantitatively, as for the four piezo fingers, the nonlinear hysteresis ratios of the lateral output displacements are within 3.95%, which is benefited from fast electrical and mechanical responses of piezoelectric ceramic. This feature helps the PRH to achieve high precision dynamic motion manipulation.

In order to investigate the displacement resolution (minimum achievable displacement) of the piezo fingers, we set the exciting signal as a stepping signal with ten equal increments and ten equal decrements during one second. We set the voltage increment and decrement as several volts and tested the displacement to see if it could produce stable displacement increments and decrements. By gradually reducing the voltage change to one step of 0.5 V, the displacement increments and decrements were still stable (Supplementary Fig. 4c). Then the average of the ten increments was calculated to evaluate displacement resolution of the piezo fingers (Supplementary Fig. 4d). These results indicate that the displacement resolutions of the four piezo fingers are within 15.45 nm.

In view of that a significant advantage of piezoelectric ceramic is fast response, the piezo finger constructed on this material can inherit fast response ability resultantly. We chose the bending motions of one finger as an example to evaluate fast response ability. The saw-tooth signals with voltage of 600 $V_{p\text{-}p}$, frequency of 1 Hz, symmetry of 0% and 100% were applied to stimulate lateral motions of the piezo finger in two directions, respectively. Then the response displacements were obtained (Supplementary Fig. 4e). These results indicate that the response time to produce maximum displacement of more than 20 μm is within 0.5 ms. According to the classical kinematic law, we can estimate that the response acceleration reaches about 160 m/s^2.

A Doppler laser vibration testing system was used to acquire the vibration characteristics of the piezo fingers (Supplementary Fig. 4f). The tested results show that the first-order natural frequencies of the four piezo fingers are 4.06 kHz, 4.09 kHz, 4.06 kHz and 4.10 kHz, respectively. These results fully reveal that the piezo fingers hold features of high natural frequency and high stiffness characteristics, which help the PRH to adapt requirements of large carrying load and high manipulation frequency.

To sum up, for the lateral bending motions of the piezo fingers, we find that they hold the significant characteristics including multi-dimensional motion, low hysteresis, high resolution, fast response and high natural frequency. Thus, the overall PRH holds these characteristics resultantly due to the configuration integration of four piezo fingers. These fundamental characteristics obviously surpass those of the other robotic hands, which can bring many advantages in the motion manipulations.

Supplementary Note 6

Functionalized hand gestures using longitudinal motion of the piezo fingers

We can also plan other gestures by utilizing the longitudinal extending motions of the piezo fingers with combination idea (Supplementary Fig. 5): (i) Gestures 8 and 9: all fingers produce extending motions along positive direction of Z axis (labeled as +Z, Supplementary Fig. 5a) and contracting motions along negative direction of Z axis (labeled as –Z, Supplementary Fig. 5b), the motions of all fingertips along Z axis are achieved resultantly. (ii) Gesture 10: the fingers 1 and 2 contract along –Z, while the fingers 3 and 4 extend along +Z (Supplementary Fig. 5c). (iii) Gesture 11: the fingers 1 and 2 extend along +Z, while the fingers 3 and 4 contract along –Z (Supplementary Fig. 5d), which are just the reverse situations of gesture 10. (iv) Gesture 12: the fingers 1 and 4 contract along –Z, while the fingers 2 and 3 extend along +Z (Supplementary Fig. 5e). (v) Gesture 13: the fingers 1 and 4 extend along +Z, while the fingers 2 and 3 contract along –Z (Supplementary Fig. 5f), which are just the reverse situations of gesture 12. The abovementioned functionalized hand gestures will bring more flexible motion manipulation ability of our PRH.

Supplementary Note 7

Description for noise experiments of the PRH

Good man-machine compatibility is a factor worth considering, but the working frequency of the PRH at hundreds of Hertz may produce working noise due to the fast excitation of the piezoelectric ceramic components. Thus, we carried out noise experiment of the PRH under maximum working voltage of 600 $V_{p\text{-}p}$ and different working frequencies, in which a digital noise meter is used to capture the noise level of the PRH when working (Supplementary Fig. 8c). The tested results show that the noise level of the PRH is less than 53.74 dB under maximum working frequency of 270 Hz and less than 57.42 dB under the maximum tested frequency of 360 Hz (Supplementary Fig. 4g). The working noise 53.74 dB under the maximum working frequency of 270 Hz is within acceptable level of human ear, just liking the noise level of loud talking (far away from the general endurable limit 100 dB of human ear). It is worth noting that the potential working scenario of our PRH is intermittent high-precision motion manipulation, and its working frequency can be set as low level, which enables us to maintain the noise level within the comfortable range.

Supplementary Note 8

Description for thermal characteristic experiments

In order to evaluate the heating level of the PRH when working, we carried out a thermal characteristic experiment. A thermal imager (Model: UTI380, UNI-Trend Technology (China) Co., Ltd, China) and a laser displacement sensor (Model: LK-H020, Keyence Co. Ltd, Japan) are utilized to capture the thermal image and response displacement of the PRH (Supplementary Fig. 8d). The PRH is continuously excited with the maximum working voltage of 600 $V_{p\text{-}p}$ and the maximum working frequency of 270 Hz for more than 60 min, and the temperature and the response displacement are measured by time interval of 5 min. The tested results show that the temperature of the piezo finger (part of piezoelectric rings and part of fingertip) keeps no

obvious change with continuous working time of 60 min, and the response displacement on the fingertip is also unchanged (Supplementary Fig. 4h and Fig. 4i). The thermal images of the PRH under different working time are shown in Supplementary Fig. 4j to Fig. 4m. These experiments reflect that our PRH has no heating phenomenon when working continuously with the maximum working voltage and frequency. This helps to ensure the stability of its own characteristics and avoid the influence of the PRH on the manipulated objects. The temperature around the piezo finger is unchanged when working, which means that the response characteristics including the response speed and amplitude of the piezo finger cannot be affected by the nonexistent heating problem. Thus, the operated samples of the PRH are also not affected by the heating problem.

Supplementary Note 9

Characteristic comparison between the plate manipulated with the PRH and other precision stages

Manipulating the plate to produce two translational DOFs and one rotary DOF motions is an important capability of our PRH, which is experimentally evaluated in detail in this work. In order to further evaluate the level of its manipulation characteristics, a simple characteristic comparison between the motion plate manipulated with our PRH and other precision stages is accomplished (Supplementary Table 3). The compared items contain the motion DOF, principle, overall size, motion stroke, working voltage, working frequency and load capability. The compared results show that the motion plate manipulated with our PRH holds several merits: (i) the motion plate manipulated with our PRH achieves greater motion strokes; (ii) the working frequency is more than other motion stages; (iii) the load capability of the motion plate manipulated with our PRH achieves excellent capability to carry other objects for motions. It is worth noting that the plate manipulated with our PRH is only a construction case of many

promising functions. In the follow-up work, we will also consider using the PRH to build multi-DOF devices for specific applications.

Supplementary Note 10

Experimental system and measurement method

We used an xPC system (Supplementary Fig. 8a) to acquire the displacements of the fingertips and the manipulated plate in the characteristic experiments. In detail, the exciting signals were generated by the programs in the host PC and converted to analog signals by a 16-bits D/A converter (PCI-1721, Advantech Co, Ltd. China), and the analog exciting signals were applied on the power amplifier (E00.A3, Harbin Core Tomorrow Science & Technology Co. Ltd. China). Then the amplified signals were used to excite the PRH. The displacements on the fingertips were acquired by the capacitive sensor (D-E20.200, Physik Instrumente (PI), Germany), and the acquired displacement signals were converted to the digital signals by a 16-bits A/D converter (PCI-1716, Advantech Co, Ltd. China), and further uploaded to the host PC for data processing. It should be noted that the displacements of the manipulated square plate were measured by laser displacement sensors (LK-H020, Keyence, Japan). Specifically, when the laser heads were used to measure the motion displacements of the square plate (Supplementary Fig. 8b), the linear displacement was obtained directly, while the rotary displacement was approximately obtained by using two laser heads:

$$\theta = \arctan\frac{|x_1| + |x_2|}{L_\mathrm{p}} \tag{7}$$

where θ is the rotary displacement of the square plate around Z_p axis; x_1 and x_2 denote the measured displacements acquired by the two laser heads; L_p is the distance between the two laser spots emitted by two laser heads. Note: this indirect method to obtain rotary displacement is only suitable for the small rotary angle.

Supplementary Table 1

Key quantitative manipulating characteristics of the plate P1

Items	LX_p DOF	LY_p DOF	RZ_p DOF
**Maximum no-load manipulating velocity**	5912.60 μm/s	6000.01 μm/s	382.47 mrad/s
**Manipulating velocity with carrying load of 0 kg at frequency of 1 Hz**	18.73 μm/s	19.74 μm/s	1.57 mrad/s
**Manipulating velocity with carrying load of 14.76 kg at frequency of 1 Hz**	4.35 μm/s	5.75 μm/s	1.18 mrad/s
**Velocity decay rate with carrying load of 14.76 kg**	76.78%	70.87%	24.84%
**Stuck carrying load with zero manipulating velocity (by estimation)**	19.22 kg	20.83 kg	59.42 kg
**The ratio between the stuck carrying load and the self-weight of the PRH**	49.28 times	53.41 times	152.36 times

Supplementary Table 2

Shapes, materials and dimensions of the selected ten manipulated objects

Object labels	Shapes	Materials	Overall dimensions
**P1**	Flatbed	Stainless steel	65 mm × 65 mm × 3 mm
**P2**	Flatbed	Acrylic	100 mm × 100 mm × 8 mm
**P3**	Flatbed	Iron	70 mm × 70 mm × 30 mm
**C1**	Cylindrical	Stainless steel	Φ65 mm × 55 mm
**C2**	Cylindrical	Polypropylene film	Φ110 mm × 60 mm
**C3**	Cylindrical	Bearing steel	Φ320 mm × 48 mm
**S1**	Spherical (hollow)	Stainless steel	SΦ300 mm × 2 mm (thickness)
**S2**	Spherical	Bearing steel	SΦ60 mm
**S3**	Spherical	Bearing steel	SΦ70 mm
**S4**	Spherical	Glass	SΦ100 mm

Supplementary Table 3

Characteristic comparison between the plate manipulated with the PRH and other precision stages

Item	Compliant platform[1]	Micro-positioning stage[2]	Nano-positioning stage[3]	Nano-positioning stage[4]	Nano-positioning platform[5]	Micro/nano positioning stage[6]	Plate manipulated with the PRH
**DOF**	2R	3L	2L	2L	3L+3R	2L	2L+1R
**Principle**	Piezo	Piezo	Piezo	Piezo	Electromagnetic	Electromagnetic	Piezo
**Overall size**	30×41×41mm^3	77×77×77mm^3	NA	NA	250×250×57.4mm^3	NA	ϕ85mm×124mm
**Motion stroke**	2.04mrad ×2.12mrad	582μm ×517μm ×524μm	1.035mm×1.035mm	42μm×42μm	0.5mm×0.5mm×5mrad	2.13mm×2.02mm	25mm×25mm ×2πrad
**Working voltage**	100V	100V	150V	150V	48V	NA	600V_{p-p}
**Working frequency**	100Hz	69Hz	32Hz	100Hz	<50.8Hz	<43.7Hz	270Hz
**Load capability**	0.25kg	NA	0.1kg	NA	0.9kg	2kg	14.76kg

Notes: “NA” means the compared item is not available.

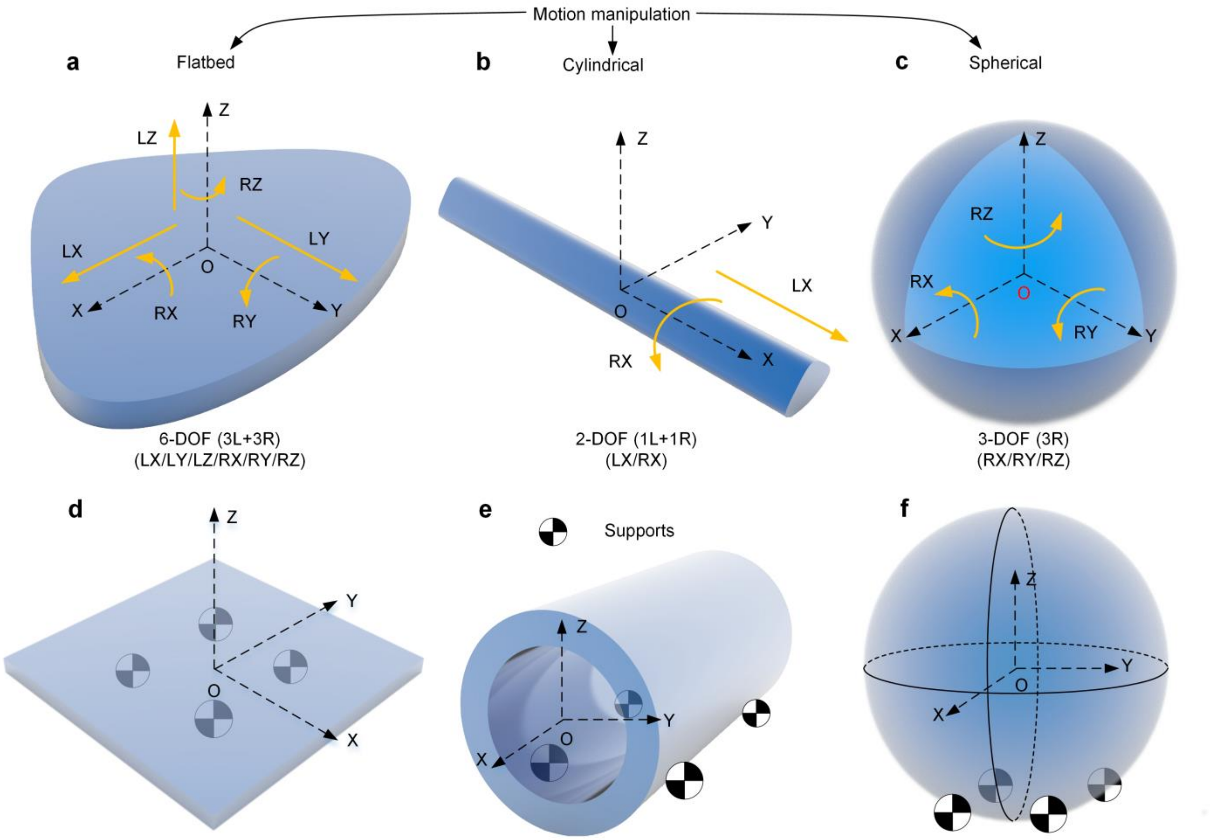

Supplementary Fig. 1.

Motion forms and supporting strategy of flatbed, cylindrical and spherical objects. **a** 3-DOF linear motions and 3-DOF rotary motions of the flatbed object. **b** 1-DOF linear motion and 1-DOF rotary motion of the cylindrical object. **c** 3-DOF rotary motions of the spherical object. **d** Supporting for a plate. **e** Supporting for a hollow cylinder. **f** Supporting for a sphere. Note: LX, LY and LZ represent linear motions along X, Y and Z axes, respectively; RX, RY and RZ indicate rotary motions around X, Y and Z axes, respectively.

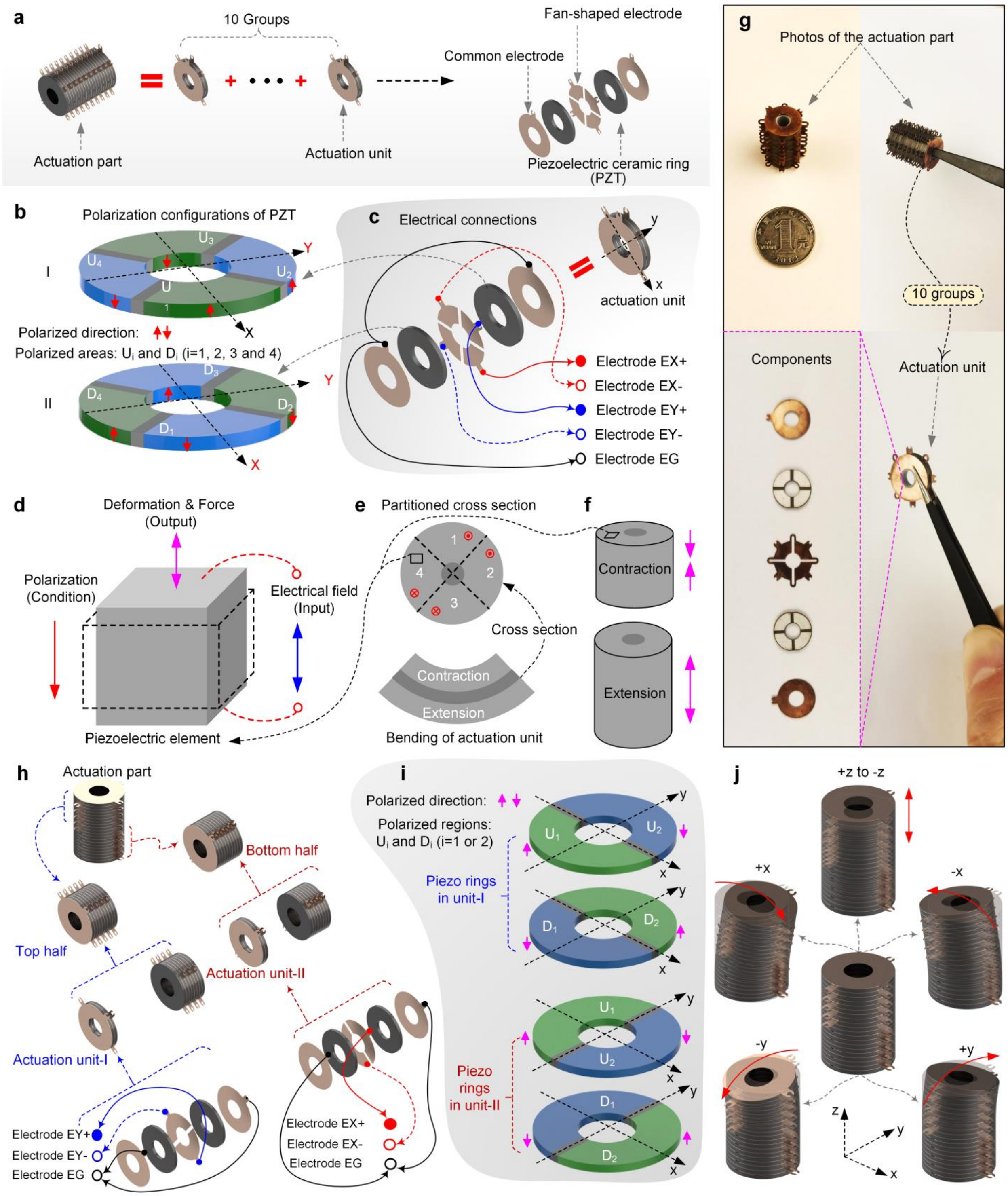

Supplementary Fig. 2.

Configurations and deformation principles of the actuation part. **a** Detailed configurations of the actuation part. **b** Polarization configurations of the two adjacent piezoelectric ceramic rings in one

group of actuation unit. **c** Electrical connection relationships of the actuation unit. **d** Diagram of the inverse piezoelectric effect of the piezoelectric ceramic. **e** Diagram of bending deformations of the actuation part. **f** Diagram of extending deformation of the actuation part. **g** Photos of the actuation part and its components. **h** Structural configurations and electrode definitions of another alternative scheme of the actuation part. **i** Structural configurations of adjacent two piezo rings in the alternative scheme. **j** Three-dimensional deformations of the alternative scheme.

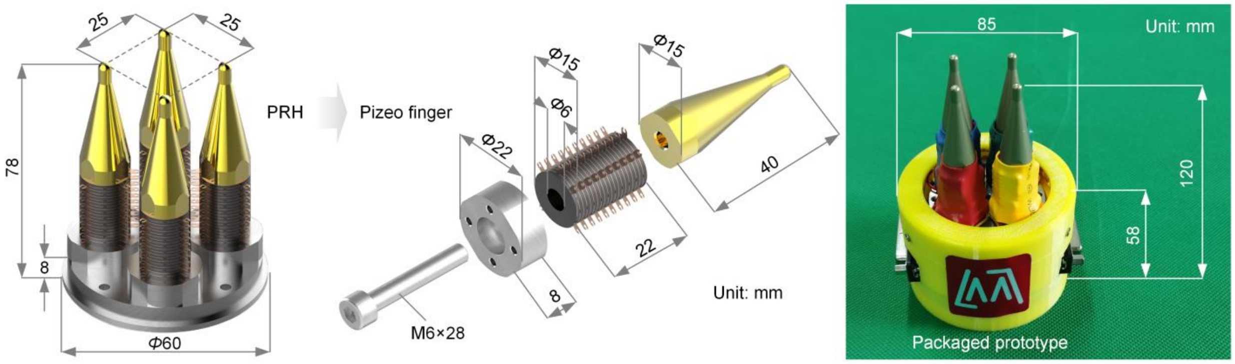

Supplementary Fig. 3.

Key structural parameters of the PRH and its packaged prototype. Note: each piezoelectric ceramic ring is with outer diameter of 15 mm, inner diameter of 6 mm and thickness of 1 mm.

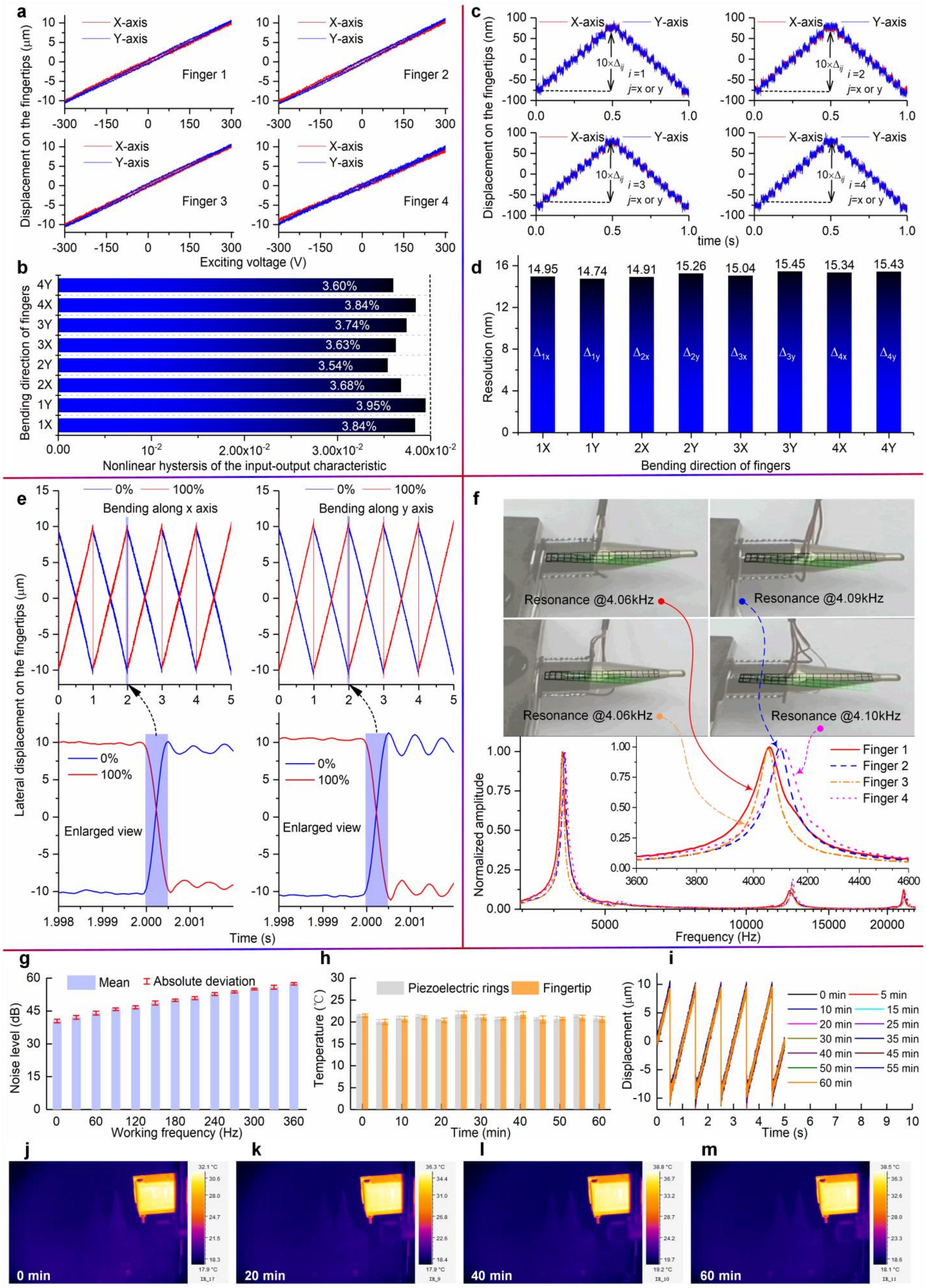
a
X-axis
Y-axis
Finger 1
Finger 2
Finger 3
Finger 4
Displacement on the fingertips (μm)
Exciting voltage (V)
b
4Y 3.60%
4X 3.84%
3Y 3.74%
3X 3.63%
2Y 3.54%
2X 3.68%
1Y 3.95%
1X 3.84%
Bending direction of fingers
Nonlinear hysteresis of the input-output characteristic
c
10×Δij
i =1 j=x or y
i =2 j=x or y
i =3 j=x or y
i =4 j=x or y
Displacement on the fingertips (nm)
time (s)
d
14.95 14.74 14.91 15.26 15.04 15.45 15.34 15.43
Δ1x Δ1y Δ2x Δ2y Δ3x Δ3y Δ4x Δ4y
Resolution (nm)
1X 1Y 2X 2Y 3X 3Y 4X 4Y
Bending direction of fingers
e
0% 100%
Bending along x axis
Bending along y axis
Enlarged view
Lateral displacement on the fingertips (μm)
Time (s)
f
Resonance @4.06kHz
Resonance @4.09kHz
Resonance @4.06kHz
Resonance @4.10kHz
Finger 1
Finger 2
Finger 3
Finger 4
Normalized amplitude
Frequency (Hz)
g
Mean
Absolute deviation
Noise level (dB)
Working frequency (Hz)
h
Piezoelectric rings
Fingertip
Temperature (℃)
Time (min)
i
0 min 5 min 10 min 15 min 20 min 25 min 30 min 35 min 40 min 45 min 50 min 55 min 60 min
Displacement (μm)
Time (s)
j
0 min
k
20 min
l
40 min
m
60 min

Supplementary Fig. 4.

Characteristics of the piezo fingers. **a** Input-output characteristics: output displacements on the fingertips versus exciting voltages in x axis and y axis of finger 1, finger 2, finger 3 and finger 4, respectively. **b** Nonlinear hysteresis of piezo fingers. **c** Stepping displacement responses: output displacements on the fingertips in x axis and y axis of finger 1, finger 2, finger 3 and finger 4 under stepping excitation, respectively. **d** Displacement resolutions of the piezo fingers. **e** Fast response characteristics in lateral motions of the piezo finger 1: output displacements in x axis and y axis of finger 1 excited by saw-tooth signal with symmetry of 0% and 100%, respectively. **f** Vibration characteristics of the piezo fingers; note: the vibration characteristics in two directions of the same finger are identical due to the symmetrical structures. **g** Noise level of the PRH under different working frequency. **h** Temperature of the piezoelectric rings and fingertip of finger 1 under working time of 0 min to 60 min. **i** Response displacements of the piezo finger under different working time. **j, k, l** and **m** Thermal images of the PRH when working for 0 min, 20 min, 40 min and 60 min, respectively. Note: the error bars in **g** and **h** represent the measurement deviation of five repeated tests.

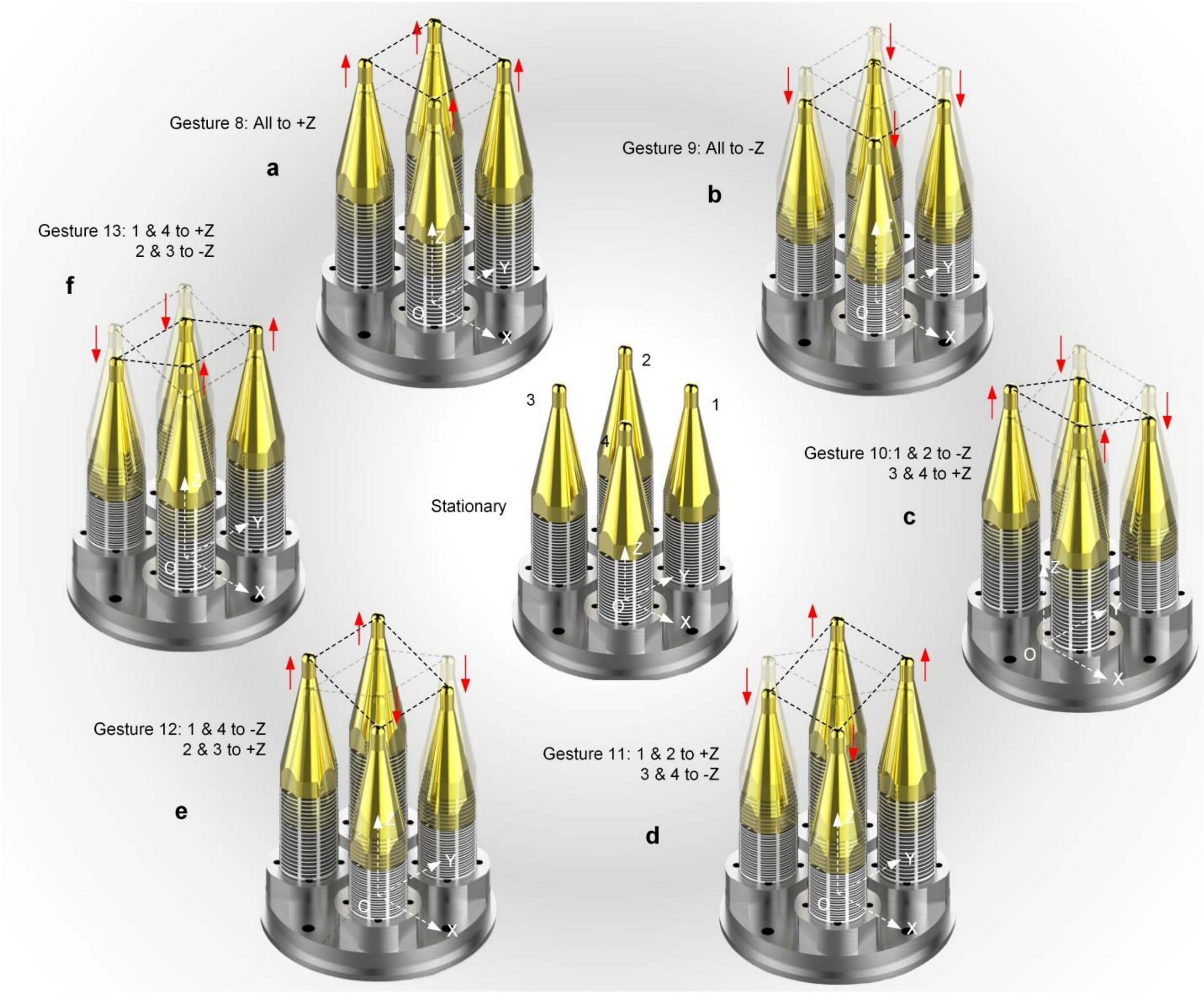

Supplementary Fig. 5.

Functionalized hand gestures using longitudinal motions of the piezo fingers. **a** and **b** Gesture 8 and gesture 9: all fingers extend along positive and negative directions of Z axis, respectively. **c** Gesture 10: the fingers 1 and 2 contract along negative direction of Z axis, meanwhile the fingers 3 and 4 extend along positive direction of Z axis. **d** Gesture 11: the reverse situation of **c**. **e** Gesture 12: the fingers 2 and 3 extend along positive direction of Z axis, meanwhile the fingers 1 and 4 contract along negative direction of Z axis. **f** Gesture 13: the reverse situation of **e**. Note: the gestures 9, 11 and 13 are just the reverse situations of gestures 8, 10, and 12, respectively.

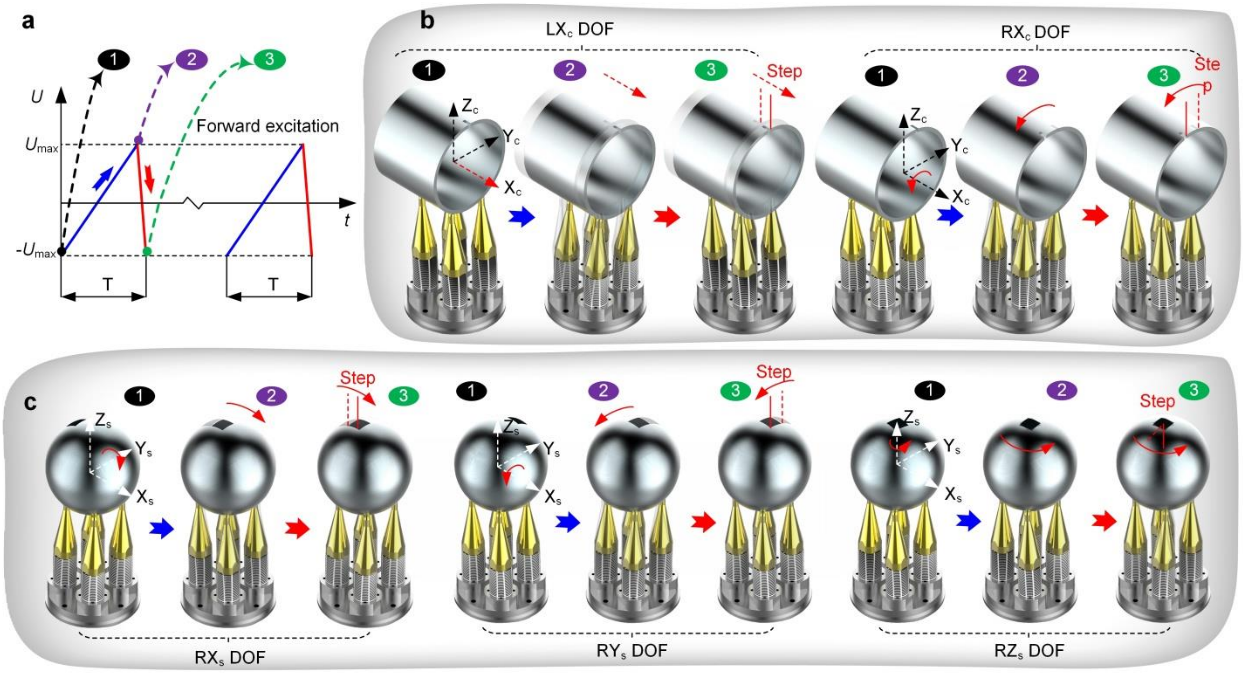

Supplementary Fig. 6.

Manipulation mechanisms of the cylindrical and spherical objects. **a** Diagram of the exciting signal used for forward manipulation. **b** Manipulation processes in one step of the linear motion in axial direction (labeled as LX_c DOF) and the rotary motion in circular direction (labeled as RX_c DOF) of the cylindrical object. **c** Manipulation processes in one step of the three rotary motions (labeled as RX_s DOF, RY_s DOF, and RZ_s DOF, respectively) of the spherical object around X_s, Y_s, and Z_s axes. Note: these manipulation processes are similar to that of manipulating plate; the local coordinate systems $X_cY_cZ_c$ and $X_sY_sZ_s$ are defined on the cylindrical and spherical objects to describe their multi-DOF motions.

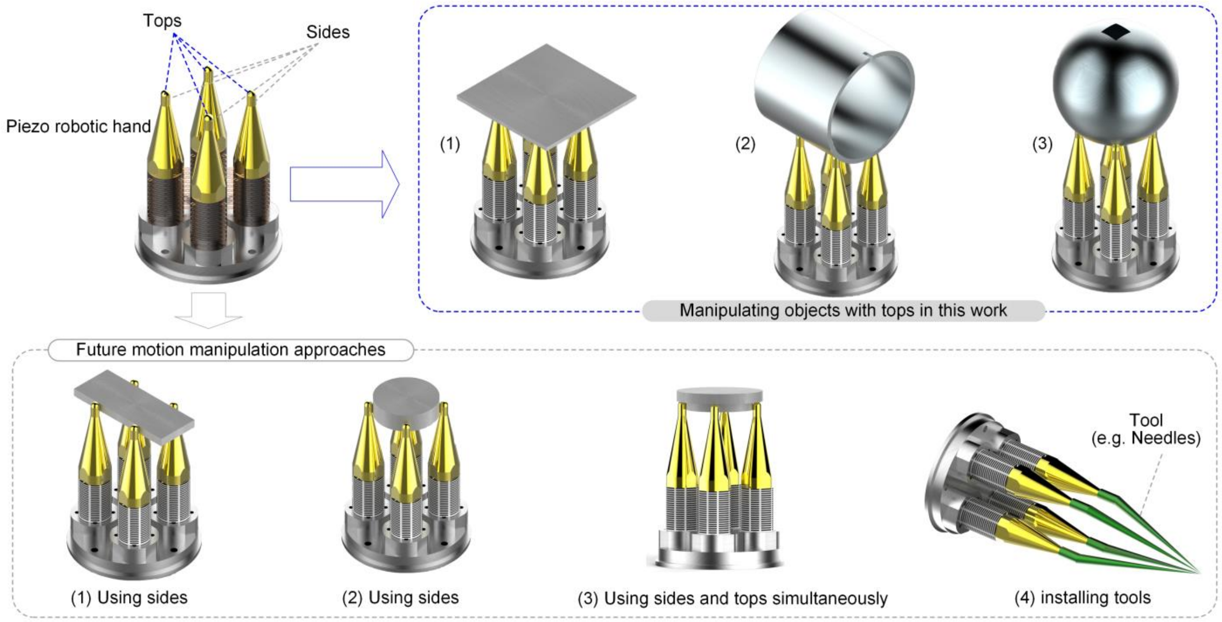

Supplementary Fig. 7.

Diagrams of motion manipulations by using sides of fingertips or installing tools.

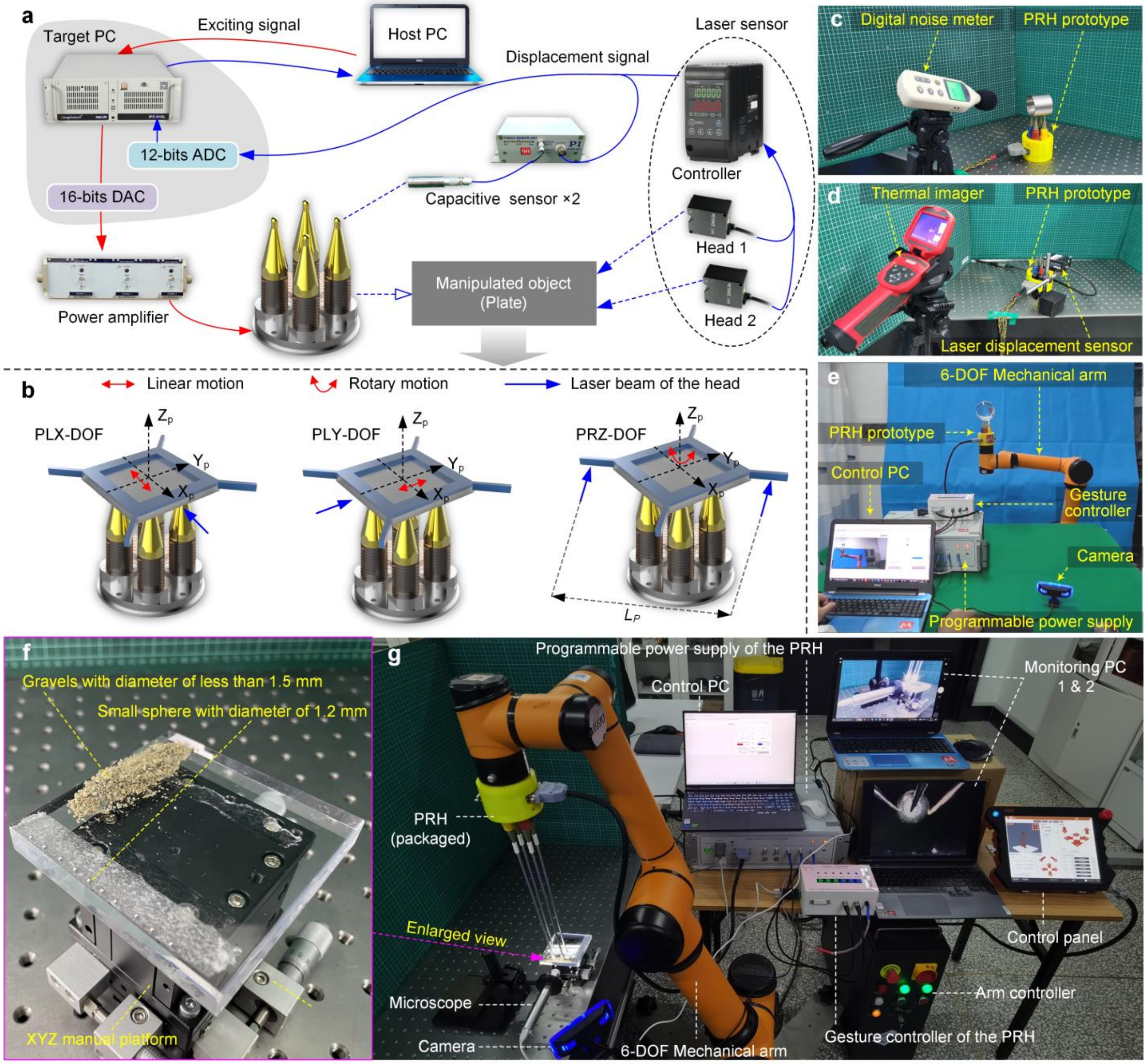

Supplementary Fig. 8.

Diagrams of the experimental setup and the measurement method. **a** Experimental setup used to acquire displacements. **b** Measurement method of measuring linear and rotary displacements of the square plate. Note: L_p is the distance between the two sensors when measuring rotary displacements; the part with deep blue is placed on the plate to facilitate the measurement of motion displacement, avoiding arrangement interference of the sensors. **c** Experimental setup for testing working noise level. **d** Experimental setup for testing thermal characteristics. **e** Experimental setup

for combination application of mechanical arm and the PRH. **f** Photos of the small spheres and gravels placed on a XYZ Platform. **g** Experimental setup for grasping experiments.

Supplementary references

1. Liang CM, *et al.* A 2-DOF Monolithic Compliant Rotation Platform Driven by Piezoelectric Actuators. *Ieee T Ind Electron* **67**, 6963-6974 (2020).

2. Zhang XZ, Xu QS. Design and testing of a new 3-DOF spatial flexure parallel micropositioning stage. *Int J Precis Eng Man* **19**, 109-118 (2018).

3. Tang H, Li JD, Jia YJ, Gao J, Li YM. Development and Testing of a Large-Stroke Nanopositioning Stage With Linear Active Disturbance Rejection Controller. *Ieee T Autom Sci Eng* **19**, 2461-2470 (2022).

4. Chen Z, Shi JJ, Li ZP, Zhong XN, Zhang XM. A Damped Decoupled XY Nanopositioning Stage Embedding Graded Local Resonators. *Ieee-Asme T Mech* **27**, 256-267 (2022).

5. Kang S, Lee MG, Choi YM. Six Degrees-of-Freedom Direct-Driven Nanopositioning Stage Using Crab-Leg Flexures. *Ieee-Asme T Mech* **25**, 513-525 (2020).

6. Cai KH, Tian YL, Liu XP, Zhang DW, Shang JK, Shirinzadeh B. Development and control methodologies for 2-DOF micro/nano positioning stage with high out-of-plane payload capacity. *Robot Cim-Int Manuf* **56**, 95-105 (2019).
