# Supplementary material for: Piezo robotic hand for motion manipulation from micro to macro
Source: Nat Commun. 2023 Jan 30;14:500. doi: 10.1038/s41467-023-36243-3 (PMC9887007; doi:10.1038/s41467-023-36243-3)
Supplement: Supplementary file 3 — Description of Additional Supplementary Files [file 41467_2023_36243_MOESM3_ESM.pdf]

Inventory of Supporting Information

1. **Supplementary information file**
2. **Supplementary movies as follows:**

Supplementary Movie 1: Multi-dimensional motions of the piezo finger.

Supplementary Movie 2: Functionalized hand gestures of the PRH.

Supplementary Movie 3: Multi-dimensional micro motions on the top of the fingertips.

Supplementary Movie 4: Multi-DOF motion manipulation mechanisms of various objects.

Supplementary Movie 5: Experimental records of 3-DOF motion manipulation of plate P_1 with carrying load of 14.76 kg.

Supplementary Movie 6: Experimental records of 3-DOF motion manipulation of plates P_1, P_2 and P_3.

Supplementary Movie 7: Experimental records of manipulating cylindrical objects and spherical objects.

Supplementary Movie 8: Experimental records of the three application scenarios.

Supplementary Movie 9: Experimental records of applications of the integrated PRH system.

Supplementary Movie 10: Experimental records of grasping applications of the PRH system.
